# Prokaryotic Chaperonins as Experimental Models for Elucidating Structure-Function Abnormalities of Human Pathogenic Mutant Counterparts

**DOI:** 10.3389/fmolb.2016.00084

**Published:** 2017-01-09

**Authors:** Everly Conway de Macario, Frank T. Robb, Alberto J. L. Macario

**Affiliations:** ^1^Department of Microbiology and Immunology, School of Medicine, University of Maryland at Baltimore, Columbus Center; Institute of Marine and Environmental TechnologyBaltimore, MD, USA; ^2^Euro-Mediterranean Institute of Science and TechnologyPalermo, Italy; ^3^Institute for Bioscience and Biotechnology Research, University of Maryland, College ParkRockville, MD, USA

**Keywords:** chaperonopathies, Group II chaperonins, experimental models, archaea, CCT-like chaperonin in bacteria, CCT5 mutations, *Pyrococcus furiosus*, hexadecamer instability

## Abstract

All archaea have a chaperonin of Group II (thermosome) in their cytoplasm and some have also a chaperonin of Group I (GroEL; Cpn60; Hsp60). Conversely, all bacteria have GroEL, some in various copies, but only a few have, in addition, a chaperonin (tentatively designated Group III chaperonin) very similar to that occurring in all archaea, i.e., the thermosome subunit, and in the cytosol of eukaryotic cells, named CCT. Thus, nature offers a range of prokaryotic organisms that are potentially useful as experimental models to study the human CCT and its abnormalities. This is important because many diseases, the chaperonopathies, have been identified in which abnormal chaperones, including mutant CCT, are determinant etiologic-pathogenic factors and, therefore, research is needed to elucidate their pathologic features at the molecular level. Such research should lead to the clarification of the molecular mechanisms underlying the pathologic lesions observed in the tissues and organs of patients with chaperonopathies. Information on these key issues is necessary to make progress in diagnosis and treatment. Some of the archaeal organisms as well as some of the bacterial models suitable for studying molecular aspects pertinent to human mutant chaperones are discussed here, focusing on CCT. Results obtained with the archaeon *Pyrococcus furiosus* model to investigate the impact of a pathogenic CCT5 mutation on molecular properties and chaperoning functions are reviewed. The pathogenic mutation examined weakens the ability of the chaperonin subunit to form stable hexadecamers and as a consequence, the chaperoning functions of the complex are impaired. The future prospect is to find means for stabilizing the hexadecamer, which should lead to a recovering of chaperone function and the improving of lesions and clinical condition.

## Introduction

The realization that abnormal chaperones can be determinant etiologic-pathogenic factors has revealed the need to develop means for studying the molecular mechanisms involved. A number of questions still require answers if progress is to be made in the elucidation of the mechanism of disease and thereby, in early diagnosis and treatment. For instance: What is the impact of a pathogenic mutation on the intrinsic properties (e.g., stability in the face of stress, and flexibility) of the chaperone molecule? and What is the effect of these mutations on the chaperoning and non-chaperoning functions (e.g., protection of other proteins from denaturation by stressors, dissolution of fibrillar protein deposits, assistance in the folding of a nascent polypeptide, interaction with cells of the innate immune system) of the chaperone? In order to find answers to these key questions, experimental models are necessary. In this review, we provide information on archaeal organisms and on some exceptional bacteria that carry Groups I and II (or Group II-like) chaperonin genes, which have potential as experimental models to study human chaperonopathies.

## Scope and objective

This review encompasses the emerging field that comprises the use of archaeal organisms and some recently identified bacteria with archaeal-like chaperonins for studying issues directly relevant to human Medicine. The focus is on Group II chaperonins and associated genetic chaperonopathies. The main objective is to present this novel area of research to the scientific community so interest in these serious diseases might be ignited and projections into other similar disorders might be perceived. It is hoped that innovative research on these and many other conditions with comparable etiopathogenic characteristics, involving abnormal chaperones, will be initiated applying the prokaryotic models discussed.

## Chaperone genes and associated chaperonopathies

Diseases caused by mutations in molecular chaperone genes, the genetic chaperonopathies, are being diagnosed with increasing frequency and numerous examples representing the main chaperone families have been reported. For instance, concerning the chaperonins, among the latest reports of mutations associated with disease one pertains to HSPE1 (Bie et al., 2016) and another to the *cct2* gene (Minegishi et al., 2016). It may be assumed that many more cases of this kind of diseases will be found when the medical community becomes aware of the existence and high prevalence of chaperonopathies, genetic and acquired. To gain perspective on the potential scope of chaperonopathies one may recall the number of chaperone genes that have so far been identified in the human genome: at least 17 for Hsp70 (Brocchieri et al., 2008); 14 for CCT, and one for Hsp60 (HSPD1, Cpn 60) (Mukherjee et al., 2010; Bross and Fernandez-Guerra, 2016); 10 for the sHsp with the alpha-crystallin domain, one for Hsp10 (HSPE1, Cpn10), five for Hsp90, and nearly 50 for Hsp40/DnaJ (Kappé et al., 2003, 2010; Kampinga et al., 2009; Macario et al., 2013; Bross and Fernandez-Guerra, 2016). Furthermore, one has to include the many molecules such as co-chaperones and chaperone cofactors and closest interactors-receptors that, in addition to the chaperones themselves, integrate the chaperoning system. Thus, it is certain that there are many more diseases or syndromes associated with mutations or post-translational modifications of chaperones and closely associated molecules than those that have been identified to date.

In this article, we will focus on the chaperonins of Group II, namely the CCT chaperonins, since there are reports on diseases caused by mutations of human *cct* genes. Fourteen *cct* genes, including canonical and non-canonical family members, have been identified in the human genome (Table 1; Mukherjee et al., 2010), and mutations in several of them have been found to cause heritable disease (Table 2).

**Table 1:** **The human ***hsp60***-***cct*** gene extended family^**a**^**.

**Name**	**Alternative names**	**St^b^**	**Chr**	**Ex**	**Is**	**aa**
CCT1	TCP1, CCTa, CCTα, TCP-1α	–	6q25.3	12, 7	2	556, 401
CCT2	CCTβ, TCP-1β	+	12q15	14	1	535
CCT3	CCTγ, TCP-1γ	–	1q23.1	13, 13, 12	3	545, 544, 507
CCT4	CCTδ, TCPD, TCP-1δ	–	2p15	13	1	539
CCT5	CCTε, TCP1E, TCP-1ε	+	5p15.2	11	1	541
CCT6A	CCTζ, CCTζ1, TCP-1ζ, CCT6, Cctz, HTR3, TCP20, TCPZ, TTCP20	+	7p11.2	14, 13	2	531, 486
CCT6B	CCTζ-2, TCP-1ζ-2, Cctz2, TSA303, Tcp20	–	17q12	14	1	530
CCT7	CCTη, TCP-1η, Ccth, NIP7-1	+	2p13.2	12, 7	2	543, 339
CCT8	CCTθ, TCP-1θ, Cctq	–	21q21.3	15	1	548
CCT8L1	LOC155100	+	7q36.1	1	1	557
CCT8L2	GROL, CESK1	–	22q11.1	1	1	557
MKKS	BBS6	–	20p12.2	4, 4	2	570, 570
BBS10	C12orf58, FLJ23560	–	12q21.2	2	1	723
BBS12	C4orf24, FLJ35630, FLJ41559	+	4q27	1	1	710
HSPD1	GROEL, HSP60, Hsp60, SPG13, CPN60, Cpn60, HuCHA60	–	2q33.1	11, 11	2	573, 573
PIKFYVE	CFD, FAB1, PIP5K, PIP5K3	+	2q34	5	1	224

a *Source: Mukherjee et al., 2010*.

b *St, DNA strand with positive or negative signs indicating sequenced or complementary strand respectively; Chr, chromosome location; Ex, number of exons; Is, number of isoforms or mRNA variants, in which multiple numbers indicate the number of exons in each isoform; aa, total number of amino acids encoded in the gene; PIKFYVE, Fab1_TCP sequence domain of the PIKFYVE kinase, most similar to the apical domain of CCT3, in which features refer to the domain portion of the gene/protein*.

**Table 2:** **Diseases associated with mutations in genes encoding chaperonins and in genes phylogenetically related to the CCT family^**a**^**.

**GENE/Name/HGNC/Gene ID/UniProtKB/Swiss-Prot/Accession number**	**Chaperonopathies. Mutations**
**CCT2/**1615/10576/P78371/NM_006431	OMIM: 605139. Leber congenital amaurosis (LCA): hereditary, early-onset congenital retinopathy with macular degeneration. Mutations T400P and R516H. See Minegishi et al., 2016.
**CCT4**/1617/10575/P50991/NM_006430	OMIM: ^*^605142. Distal hereditary sensory neuropathy (mutilated foot) in rat. Mutation C450Y.
**CCT5**/1618/22948/P48643/NM_012073.3	OMIM: ^*^610150; #256840. Distal hereditary sensory-motor neuropathy. Mutation H147R. See description in this article.
**MKKS**/7108/8195/Q9NPJ1/NM_018848.2 & NM_170784.1	OMIM: 04896 gene; 209900 phenotype; 236700 phenotype; BBS6 605231; McKusick-Kaufman syndrome; MKKS hydrometrocolpos syndrome; hydrometrocolpos, postaxial polydactyly, and congenital heart malformation; HMCS Kaufman-Mckusick syndrome. Y37C (604896.0003), T57A (604896.0010), and C499S (604896.0013): increased MKKS degradation and reduced solubility relative to wildtype MKKS, and the mutant H84Y (604896.0001). R155L, A242S, and G345E mutations: increased MKKS degradation only.
**BBS10**/26291/79738/Q8TAM1/NM_024685.3	OMIM: 610148 gene; 209900 phenotype; BBS10 615987; Bardet-Biedl syndrome 10; ciliopathy with obesity, retinitis pigmentosa, polydactyly, hypogonadism, and renal failure. 209900) a 1-bp insertion at residue 91 leading to premature termination 4 codons later (C91fsX95). V11G; R34P; S303FS; S311A.
**BBS12**/26648/166379/Q6ZW61/NM_152618.2	OMIM: 610683 Phenotypes OMIN: BBS12 615989 Bardet-Biedl syndrome 12. A289P; R355T; 3-BP DEL, 335TAG; 2-BP DEL, 1114TT; 2-BP DEL, 1483GA; F372fsX373.
**HSPD1**/5261/3329/P10809/NM_199440.1 & NP_955472.1	OMIM: 605280 Spastic Paraplegia 13, autosomal dominant; SPG13, V98I; Q461E. See Bross and Fernandez-Guerra, 2016.
	OMIM: 612233 Leukodystrophy, Hypomyelinating, 4; HLD4; Mitochondrial HSP60 Chaperonopathy; MitCHAP60 Disease. Mutation D29G. See Bross and Fernandez-Guerra, 2016.
**HSPE1**/5269/3336/61604.2/NM_002157.2 & NP_002148.1	OMIM: ^*^600141 Neurological and developmental disorder characterized by infantile spasms. Mutation L73F. See Bie et al., 2016.

a *Source: Macario and Conway de Macario, 2005; Mukherjee et al., 2010; Macario et al., 2013; Minegishi et al., 2016. Clinical and pathological features are described in the references cited and in the URLs shown*.

**Figure 1:**
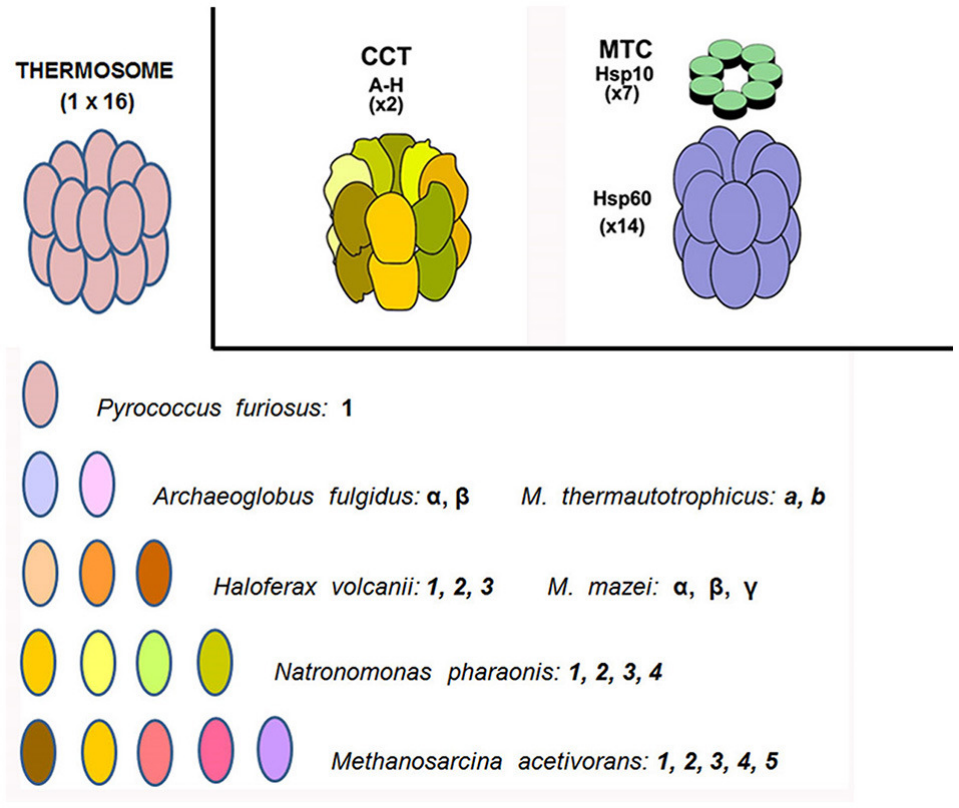
**Chaperonins of Group I and II in archaea and humans**. Top left: archaeal CCT-complex equivalent, or thermosome; top center the CCT chaperonin of Group II complex typically resident of the eukaryotic-cell cytosol; and top right the chaperonin of Group I (Hsp60 or Cpn60) complex (with Hsp10 on top) characteristic of bacteria and eukaryotic-cell mitochondria (the former two are hexadecamers while the latter is a tetradecamer). Archaeal species vary in their content of chaperonin genes-proteins from only one through a maximum (at least from what we know at the present time) of five. These subunits are variously designated with Arabic numbers, English letters, or Greek letters. As far as we know, they all form a hexadecameric megadalton sized complex, the thermosome, of the type shown on top to the left, which is an example of a hexadecamer found in archaea encoding only one chaperonin subunit, e.g., *P. furiosus*. The composition of hexadecamers in all archaea that have two or more subunits is not yet fully elucidated. *M. thermautotrophicus, Methanothermobacter thermautotrophicus* ΔH, previously known as *Methanobacterium thermoautotrophicum* ΔH. *M. mazei, Methanosarcina mazei*. Source: Macario et al., 1999, 2004; Conway de Macario et al., 2003; Maeder et al., 2005; Large and Lund, 2009.

The clinical features and mode of inheritance of genetic chaperonopathies, including those caused by mutations of chaperonin genes, are in general well characterized (see for instance Bouhouche et al., 2006a; Bross and Fernandez-Guerra, 2016). However, the molecular mechanisms causing the cellular, tissue, and organ lesions observed in patients are still poorly understood. Likewise, the impact of these pathogenic mutations on the intrinsic properties and chaperoning functions of the chaperonin molecules has not yet been characterized *in vivo* or *in vitro* to a satisfactory extent. More research and especially, more experimental models are necessary to boost progress in this area of Medicine pertaining to chaperonopathies. In this regard, prokaryotes offer interesting possibilities. All bacteria possess chaperonins of Group I and some species also have chaperonins more closely related to Group II (Techtmann and Robb, 2010). All archaea have Group II chaperonins and some also possess chaperonins of Group I (Table 3; Deppenmeier et al., 2002; Galagan et al., 2002; Conway de Macario et al., 2003; Laksanalamai et al., 2004; Maeder et al., 2005; Large and Lund, 2009). In archaea, the chaperonins of Group II are present in various modes: some have only one gene/subunit while others have two, three, four, or five genes/subunits (Figure 1). As indicated in the figure, in all cases studied so far the archaeal Group II subunits associate to form hexadecamers made of two stacked octameric rings just like the human CCT subunits do.

**Table 3:** **Examples of archaea with both, Group I and II chaperonin genes^**a**^**.

**Organism**	**Chaperonin genes of Group**:	
	**I**	**II (subunits)**
*Methanococcus vannielii* SB	1	1
*Methanospirillum hungatei* JF-1	1	2
*Methanosarcina barkeri* str. Fusaro	1	3
*Methanosarcina mazei* Go1	1	3
*Methanosarcina acetivorans* C2A	1	5

a *Source: Deppenmeier et al., 2002; Galagan et al., 2002; Conway de Macario et al., 2003; Maeder et al., 2005; Large and Lund, 2009*.

## Bacteria with Group II-like (Group III) chaperonins

Many species of Bacteria have multiple Hsp60 (Cpn60) chaperonin genes. This is especially notable in the Agrobacterium group, in the Firmicutes, and in the Alphaproteobacteria (Lund, 2009). In these bacteria, the multiple GroEL/ES homologs presumably carry out complementary protein-folding and -quality control activities and in some cases for which genetic systems are available, the essential and nonessential copies of the Cpn60 gene could be identified (Lund, 2001). In addition, the two Cpn60 homologs of *Mycobacterium tuberculosis* can act as cytokines with modulation of inflammatory responses (Qamra et al., 2005; Henderson et al., 2006, 2010). Given the predilection of *M. tuberculosis* for establishing cryptic infections, suppression of the inflammation response may be an important aspect of its molecular repertoire as a pathogen.

**Figure 2:**
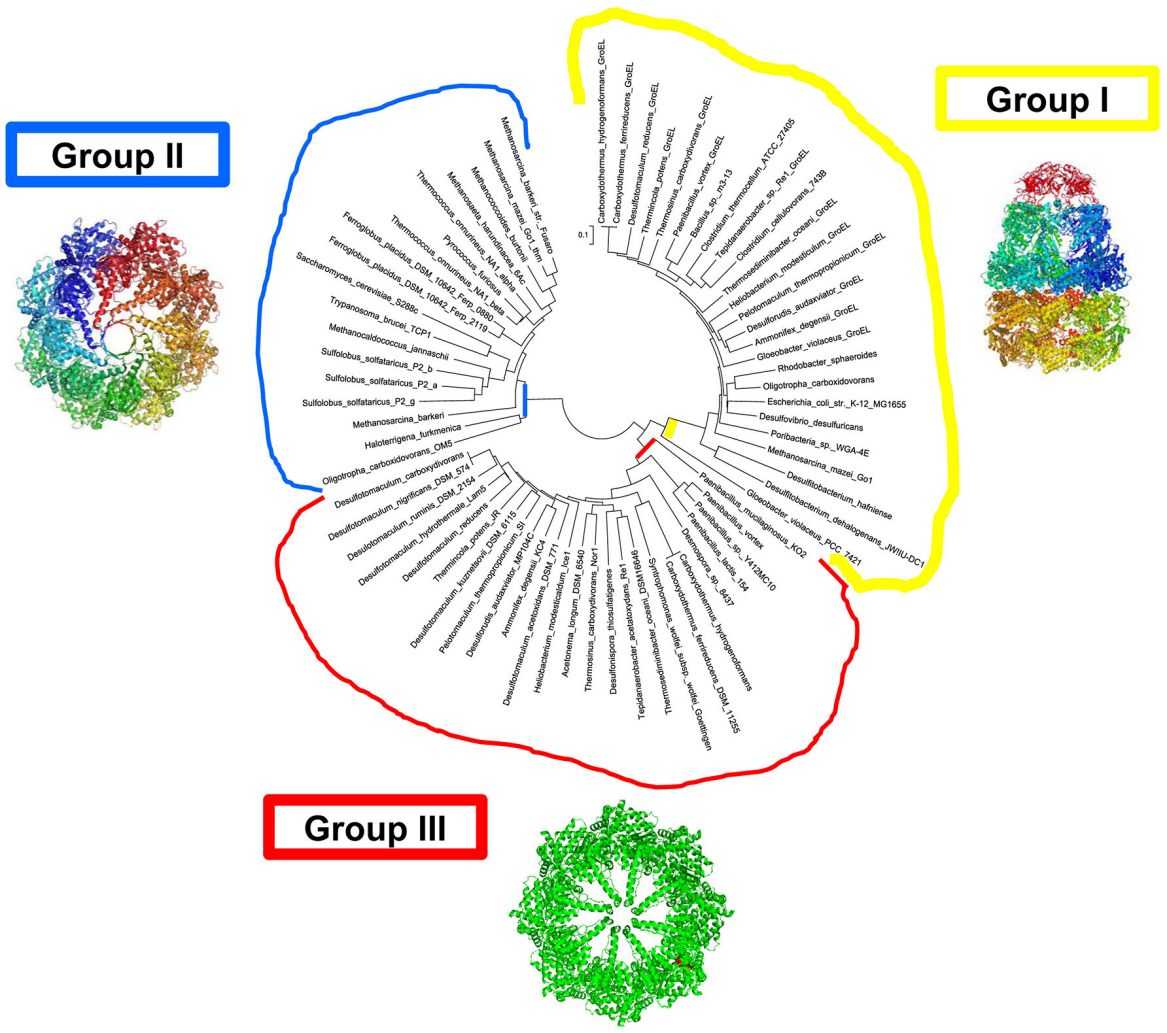
**Bacteria with chaperonins close to Group II (tentatively designated Group III)**. Molecular phylogenetic analysis by Maximum Likelihood methods. Groups I, II, and III, are in yellow, blue, and red, respectively. The evolutionary history was inferred by using the Maximum Likelihood method based on the JTT matrix-based model (Jones et al., 1992). A heuristic search was applied with Neighbor-Join and BioNJ algorithms to a matrix of pairwise distances estimated using a JTT model, and then selecting the topology with superior log likelihood value. The tree is drawn to scale, with branch lengths measured in the number of substitutions per site. The analysis involved 190 full-length chaperonin amino-acid sequences. All positions containing gaps and missing data were eliminated. There were a total of 370 positions in the final dataset. Evolutionary analyses were conducted in MEGA7. The intial alignment was constructed using ClustalW default settings.

In a group of Bacteria with taxonomic common ground, an archaeal-type chaperonin occurs in addition to the canonical GroEL/ES operons. In these organisms, with one exception, the Group II-like proteins, tentatively named Group III chaperonins (Figure 2) are coded by genes that are embedded in the DnaK(Hsp70)/DnaJ(Hsp40) operon, which encodes the Hsp70, and DnaJ(Hsp40) homologs and the nucleotide exchange factor GrpE.

Prefoldin (or Gim C), a holdase chaperone complex found in the archaeal and eukaryal sequenced genomes, interacts directly with the charged residues located in the apical domain in Group II chaperonins (Vainberg et al., 1998; Sahlan et al., 2010; Zako et al., 2016). This is a favored mode of client protein delivery to the Group II chaperonin complex for refolding. The absence of Prefoldin in bacterial genomes may imply that another chaperone is partnering with the Group III chaperonin. It is tempting to speculate that DnaK(Hsp70), which is encoded in the same operon as most chaperonin III genes, and thus likely to be coregulated, is functioning as a direct delivery mechanism, a situation observed with eukaryotic homologs. Alternatively, the Group III chaperonins may be able to function efficiently without the assistance of a co-chaperone (Cuéllar et al., 2008). The working relationship, if any, between the chaperonins of Group I and Group III in bacteria that have both, in protein homeostasis is not characterized yet. The Group III chaperonins have been shown to provide robust resistance to lethal heat shock exposures in *Escherichia coli* when expressed as a single recombinant protein (Techtmann and Robb, 2010).

**Figure 3:**
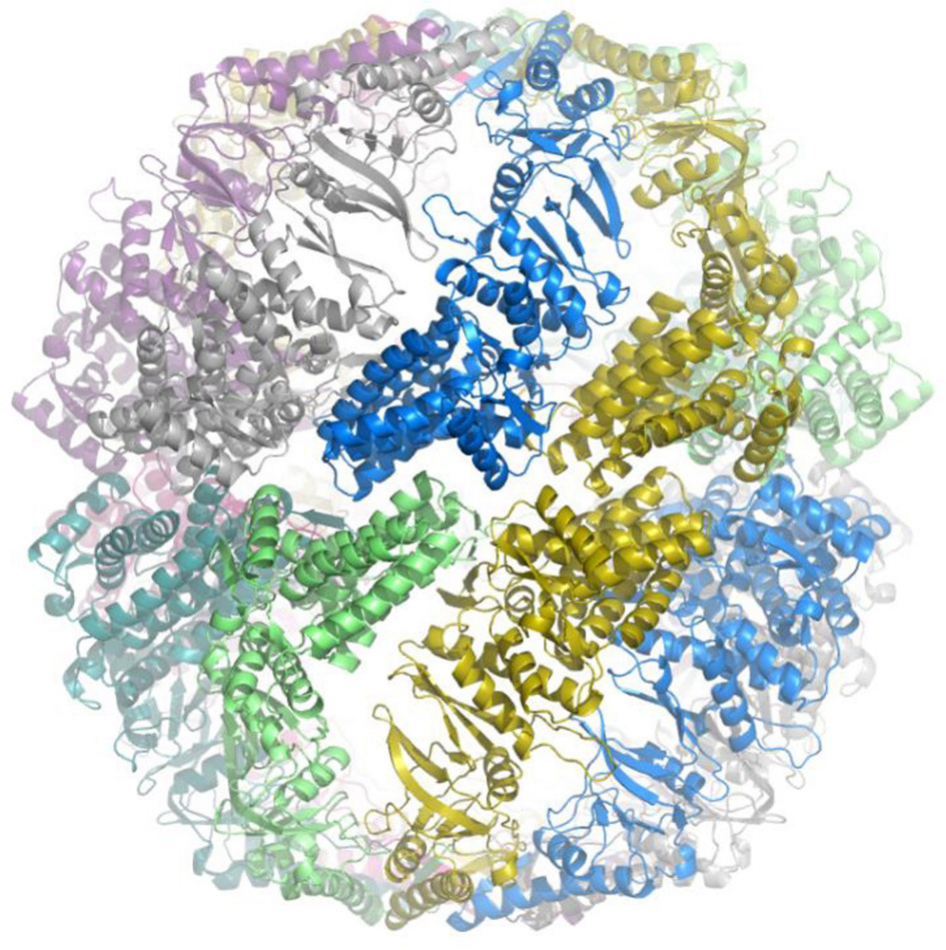
**Crystal structure of prototype of Group III chaperonin**. Crystal structure of the prototype Group III chaperonin closed complex (hexadecamer) from *Carboxydothermus hydrogenoformans*. The view is along the equatorial plane and the individual, identical subunits are coded in different colors for contrast to visualize the intersubunit contacts. The structure reveals that the inter-ring contacts are formed by one-to-one associations between the subunits in the equatorial plane and that the configuration of the apical domain (“built-in lid”) differs from the lid region of Group II chaperonin complexes. The method and preliminary structure have been described (An et al., 2016).

**Figure 4:**
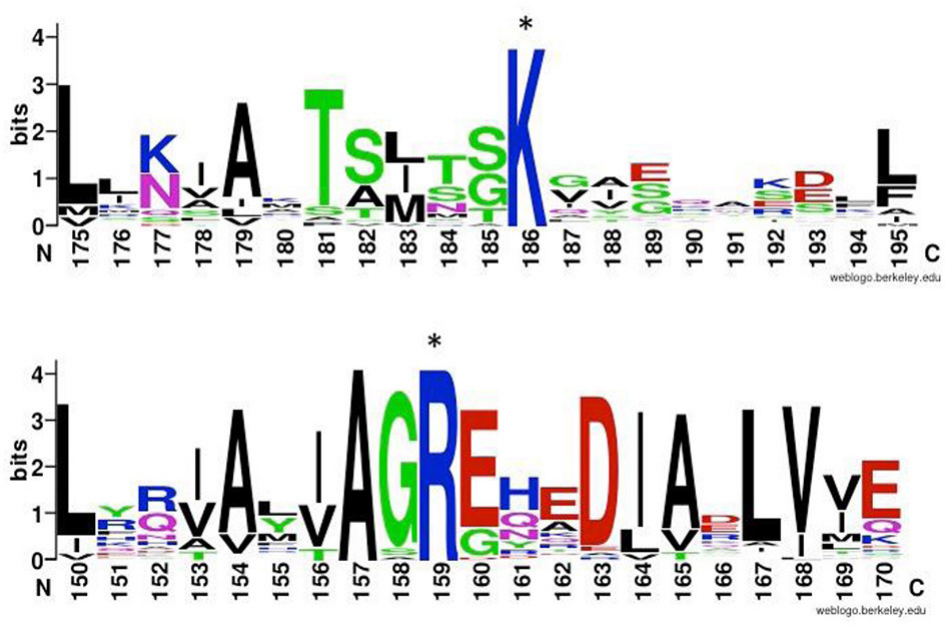
**ATP-binding sites of chaperonins of Group II and III**. Comparison of highly conserved putative ATP gamma-phosphate interacting residue (denoted by asterisks) in group II (top) and group III (bottom) chaperonins. Top: Logo created using WebLogo (Schneider and Stephens, 1990; Crooks et al., 2004) and ClustalW amino-acid sequences alignment of 26 Group II chaperonins including representatives from all eight eukaryotic subunits and archaeal sequences. Bottom: Group III chaperonins alignment of 79 sequences pulled from NCBI database by BLAST against Ch cpn amino acid sequence (Altschul et al., 1990).

The crystal structure of the prototype Group III chaperonin from *Carboxydothermus hydrogenoformans* has recently been determined (An et al., 2016), and shows distinct structural differences, which probably reveal functional differences, between the Group II and Group III chaperonins (Figure 3). Based on both the phylogenetic and functional differences in the lid domain and the nucleotide-binding cavity (Figure 4), we have proposed that this class be called the Group III Cpn60 clade (Rowland, 2016). The apical domain of the Group III chaperonin was also shown to be divergent from Group II chaperonins (Techtmann and Robb, 2010). The Group III chaperonins may be thought to represent a remnant of the common ancestor of the Group I and Group II chaperonins (Techtmann and Robb, 2010). Alternatively, the genes may have diverged from an early lateral gene transfer of a Group II chaperonin from Archaea to Bacteria. In either event, the allosteric mechanism of the Group III chaperonins is similar to the Group II with the exceptions of divergence in the apical domain and the lack of a nucleotide sensing loop.

For the purposes of testing or mimicking the biochemical effects of pathological mutations affecting, for instance, human CCT genes, the Group III chaperonins may have unique advantages. These include the ability of the prototype to complement overtaxed stress responses of bacteria such as *E. coli*, as it is expressed in soluble form and at high levels in Bacteria (Techtmann and Robb, 2010). The crystal structure that has been produced from this prototype also provides a scaffold to determine the relative structural similarity to the regions of the CCT homologs affected by pathogenic mutations (An et al., 2016).

## CCT5 pathogenic mutation

We have been working with archaeal stress proteins and chaperones for many years and are now using some archaea as experimental models to investigate molecular features of human chaperonopathies that are difficult to study directly, in human samples. Here, we will briefly discuss an archaeal experimental model that is providing interesting data pertaining to the impact of a pathogenic mutation on the properties of the chaperone molecule itself. The idea is that, by elucidating the molecular abnormalities caused by the mutation, the road will be opened to investigate the molecular mechanisms underlying the tissue lesions observed in the patients with chaperonopathies. Furthermore, biochemical and biophysical information insights that accrue from these studies will help in designing therapeutic strategies targeting the key steps of pathogenesis.

Along these lines, we are currently characterizing the molecular abnormalities involved in the causation of the pathological lesions observed in a human neuropathy associated with a mutation of the CCT5 subunit. This neuropathy was described in four members of a Moroccan family and was characterized as an autosomal recessive, mutilating, sensory neuropathy with spastic paraplegia (Bouhouche et al., 2006a,b). Onset occurred between 1 and 5 years of age and the clinical and pathological features were lower limb spasticity; hyperreflexia with clonus; positive Babinski; subtle distal amyotrophy with normal motor function; and distal sensory loss for all modalities in the upper and lower limbs, particularly in the feet. Deformities of the hands and feet were recorded in two patients, and most had deep perforating ulcers of the extremities. Progression of spasticity was slow but the sensory neuropathy was rapidly progressive and severe. Magnetic resonance imaging (MRI) of two patients showed severe atrophy of the spinal cord.

## An archaeal experimental model

Archaea have not been used as models to specifically study molecular aspects of human chaperonopathies until very recently (Min et al., 2014). However, studies on the structure and properties of the thermosome, the archaeal equivalent of the human CCT, have been going on for nearly 30 years. These studies of the thermosome have provided fundamental information useful also to understand the structure and mechanism of function of the eukaryotic counterpart. Here we will first present a few illustrative examples of structural studies of the thermosome and then, we will discuss the archaeal model we utilized to investigate molecular aspects of the chaperonopathy mentioned earlier, which is associated with mutations in the CCT5 submit.

## The structure of the archaeal thermosome

The archaeal thermosome has been the focus of study for elucidating the structure of the multisubunit chaperoning machine for many years and it continues to be a source of valuable information applicable to the human CCT complex (Waldmann et al., 1995; Ditzel et al., 1998; Bosch et al., 2000; Pereira et al., 2010, 2012; Zhang et al., 2010; Lund, 2011; Skjærven et al., 2015). For example, data supporting the notion that the archaeal thermosome is the equivalent of the eukaryotic cytosolic chaperonin CCT, also named TRiC (t-complex polypeptide 1 ring complex), were obtained over two decades ago by studying the archaeon *Thermoplasma acidophilum* (Waldmann et al., 1995). This pioneering work provided impetus to subsequent investigations using the same organism to elucidate the functions and structure of the chaperonin complex. Two thermosome subunits are encoded in the *T. acidophilus'* genome (Ruepp et al., 2000, 2001; Large and Lund, 2009) termed a and b, or alpha and beta. A crystal structure revealed that the *T. acidophilus* thermosome is composed of two stacked octameric rings composed of alternating a (alpha) and b (beta) subunits, with the two rings being related by 2-fold symmetry, which generates a-a (alpha-alpha) and b-b (beta-beta) pairs and brings about an (ab)4(ab)4 arrangement with a 42-point symmetry (Ditzel et al., 1998). The domains in the monomers follow a topology similar to that of the GroEL monomer. The combined analysis of the crystal structure and of multiple alignments of extended primary sequences of various chaperonins helped to identify in the linear sequence the now well-known three domains, as follows, from the N to the C terminus: the N-terminal segment of the equatorial domain, the N-terminal portion of the apical domain, the intermediate domain, the C-terminal segment of the apical domain, and the C-terminal segment of the equatorial domain. This was a seminal contribution useful to many researchers since it was reported. The way rings make contact is different in the archaeal thermosome as compared with the GroEL complex. Most importantly, it was observed that the apical domains extend to build a sort of lid that can close the entrance to the central cavity, playing a similar role to that of GroES in the bacterial chaperonin complex. The central cavity was found to have a polar surface which suggested its implication in the process of substrate folding.

In subsequent work, the crystal structure of the beta-apical domain was determined at 2.8 Ångström resolution (Bosch et al., 2000). A comparative analysis with previously determined apical domain structures revealed in the *T. acidophilum* a segmental displacement of the protruding part of helix H10 via the hinge GluB276-ValB278. Furthermore, the portion of the molecule including the amino acids GluB245-ThrB253 was found to be in an extended beta-like conformation in contrast to the alpha-helix characteristic of the alpha-apical domain. These conformational features prompted the suggestion that apical domain projections could be the basis to provide a variety of context-dependent conformations during an open, substrate-accepting state during the ATP-dependent chaperoning cycle.

Other structural studies with the archaeal thermosome were carried out using *Methanococcus maripaludis* (Pereira et al., 2010, 2012). The genome of *M. maripaludis* encodes only one thermosome subunit (Hendrickson et al., 2004; Large and Lund, 2009). The crystal structures of both, the open (substrate acceptor) and closed (substrate folding) states were obtained (Pereira et al., 2010). It was observed that the *M. maripaludis* thermosome subunit remains rigid during the reorientation accompanying the cycling of the complex. This is in sharp contrast with what occurs with GroEL, in which the greatest motion occurs at the intermediate and apical domains while the equatorial domain conserves a similar conformation in the open and closed states. Interestingly, the rotation occurring from the open to the closed state decreases by 65% the volume of the folding chamber whose surface becomes highly hydrophilic.

In a subsequent study with *M. maripaludis*, the mechanism responsible for communicating the local changes in the ATP-binding site between subunits during the chaperoning cycle were investigated (Pereira et al., 2012). The data obtained provided fundamental information on the movements of the thermosome components as they are engaged in the various states of the chaperoning cycle. The crystal structures of several ATP-bound states were determined and it was observed that the amino acid Lys161, known to be much conserved among Group II chaperonins, interacts with the γ-phosphate of ATP and reorients in the presence of ADP. It was found that the loss of the interaction ATP- γ-phosphate in the ADP state is accompanied by considerably rearrangement of the loop including amino acids 160–169. It was proposed that Lys161 functions as an ATP sensor and the loop is a nucleotide-sensing device whose function would be to monitor the presence of γ-phosphate. Loop mutants had considerably lower ATPase activity than the wild type molecule, which suggested that the nucleotide-sensing loop plays a critical role in synchronizing the folding cycle.

## The thermosome of *Pyrococcus furiosus*

We standardized an experimental model using the chaperonin molecule from the archaeon *Pyrococcus furiosus*. This organism has only one CCT ortholog (Robb et al., 2001) that forms an hexadecameric chaperoning machine, the thermosome, with the same overall structure as the human CCT complex, except that all 16 subunits are identical (Figure 1). Therefore, simulating a human CCT mutation on the *P. furiosus* chaperonin (Pf-Cpn) subunit would amplify the impact of the human mutation 8-fold per octameric ring, and facilitate detection of subtle functional deficiencies caused by the mutation. Mutations causing major functional deficits in human CCT are most likely lethal because the chaperonin is essential for life; one can safely assume that no phenotypes will be found associated with mutations causing major, easily detectable functional failure of the chaperonin molecule *in vitro*, or *in vivo*. Therefore, this strategy for characterizing slight deficits in function may provide valuable insights into pathogenic mechanisms. It has to be borne in mind that if the mutation in one subunit of a hetero-oligomer such as the human CCT affects primarily the interaction of the non-identical subunits with each other, the use of an homo-oligomeric model like that of *P. furiosus* may not fully report on the impact of the mutation. This consideration must be taken into account in interpreting experimental results with the two types of oligomers.

**Figure 5:**
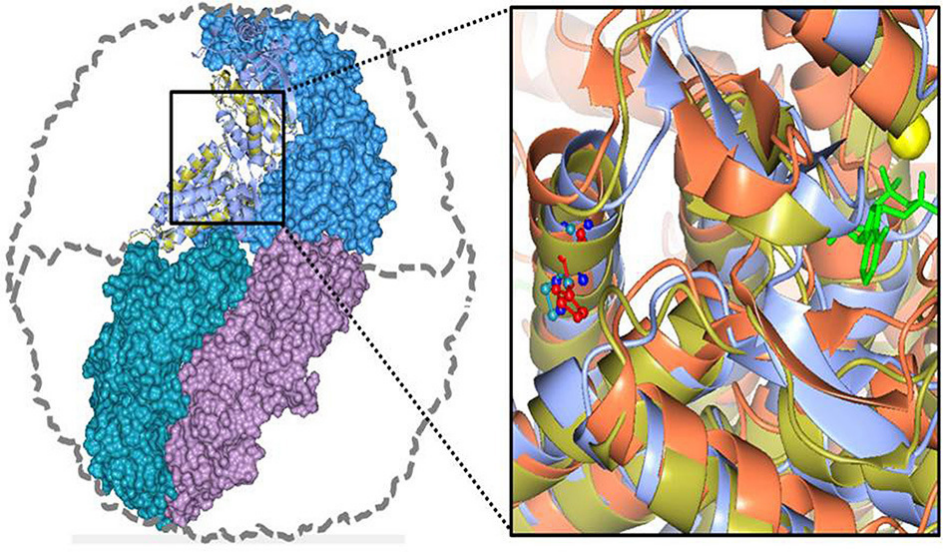
**Human CCT5 and ***P. furiosus*** Pf-Cpn (Pf-CD1) superposed onto the crystal structure of ***Thermococcus*** strain KS-1 subunit α (1Q3Q)**. Left. The Pf-CD1 graphic representation (gold ribbon) was obtained in Swiss-Model (http://swissmodel.expasy.org/) and superposed onto the crystal structure of KS-1 subunit α (monomers are displayed in marine blue, violet, and deep teal colors as surface, and in cyan color as ribbon). The whole hexadecamer double-ring structure is depicted by a dotted line. Right. Magnified image of the superposed structures of the Pf-CD1 (gold ribbon) and Human CCT5 (orange ribbon) onto the KS-1 α subunit crystal structure (1Q3Q; cyan ribbon). Side chains of isoleucine at 138 of Pf-CD1 (blue), isoleucine at 138 of KS-1 subunit α (deep teal), and histidine at 147 of human CCT5 (red) are represented as ball and stick. AMP-PNP (β,γ-Imidoadenosine 5′-triphosphate lithium salt hydrate; green stick) and magnesium ion (yellow ball). Source: Min et al., 2014.

We first established which amino acid position in the Pf-Cpn matches that in the human CCT5 in which the mutation is found, i.e., 147. In the wild type CCT5 the amino acid His is at this position but in the pathogenic mutant His is replaced by Arg. We found by a series of alignments and 3D modeling studies that position 138 in Pf-Cpn is equivalent to the 147 position in human CCT5 and it is occupied by Ile (Figure 5; information on the primary and secondary structures is available in Min et al., 2014). To examine the impact of the mutations we used Pf-CD1, which is the recombinant molecule Pf-Cpn lacking the last 22 amino acids that are involved in extreme heat resistance and have no match in the human CCTs. We introduced the pathogenic mutation Ile138Arg in Pf-CD1 to produce Pf-R, and then proceeded to test the mutant in comparison with the wild type to assess its properties and chaperoning capacity. We also made and used for comparison another mutant, not known to be pathogenic, Ile138His of Pf-CD1 (Pf-H).

**Figure 6:**
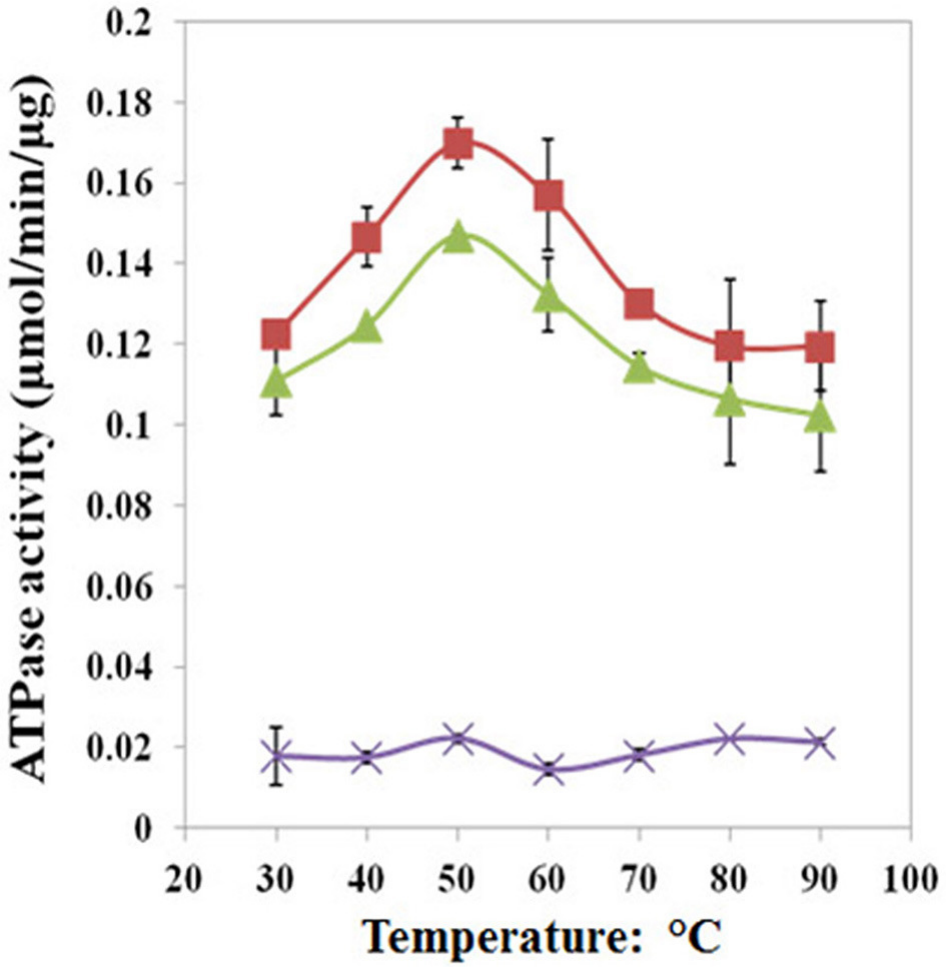
**The pathogenic mutant causes a loss of ATPase activity evidenced at various temperatures**. Pf-CD1 (red squares), Pf-H (green triangles), and Pf-R (purple stars). The results shown are mean values (± standard deviations) and the experiments were carried out in triplicate. Source: Min et al., 2014.

**Figure 7:**
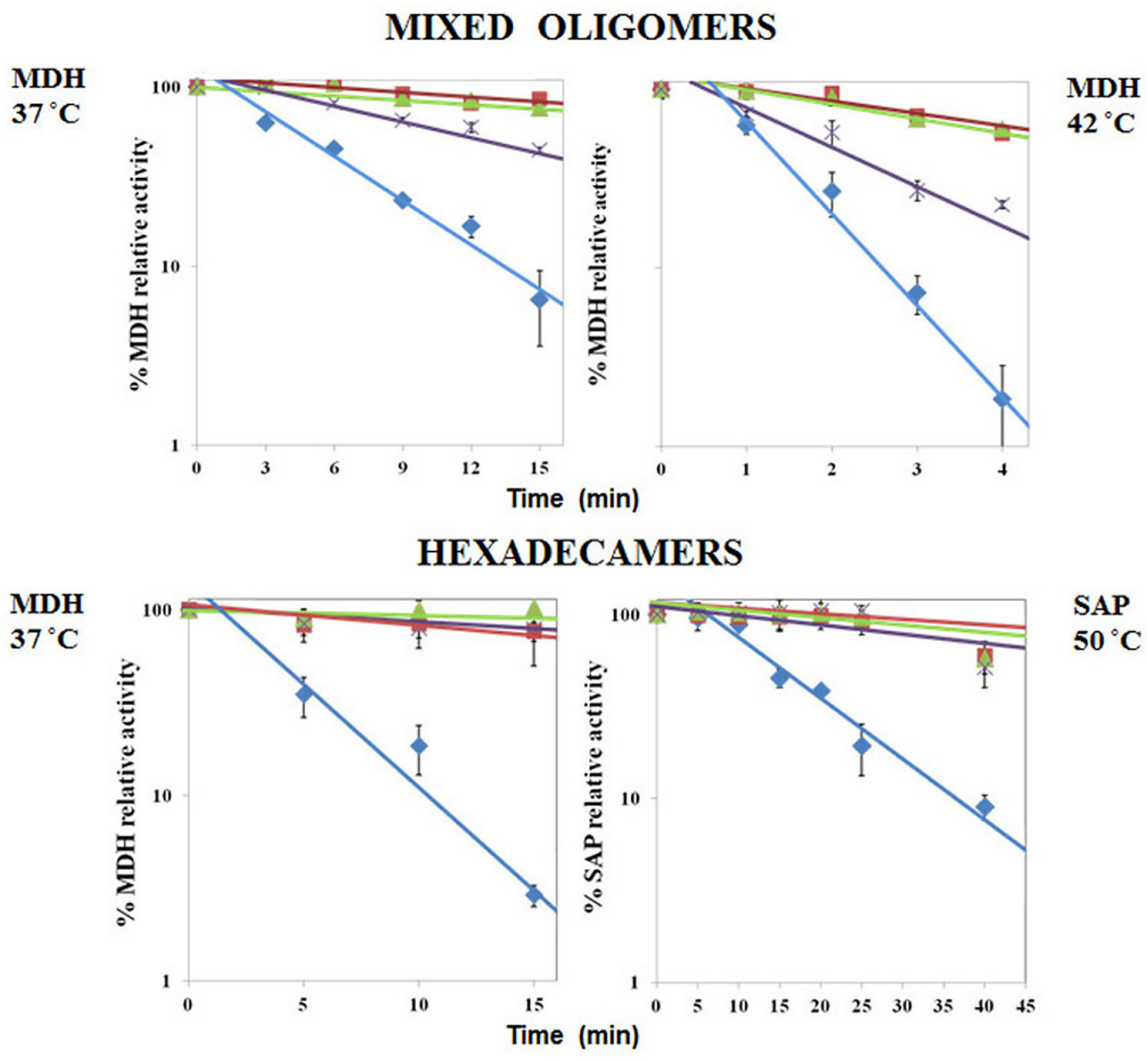
**Comparative analyses of protective capacity of mutant and wild type chaperonins**. Protection from heat denaturation of malate dehydrogenase (MDH) by mixed oligomers of Pf-CD1 (red square), Pf-H (green triangle), and Pf-R (purple star) at 37°C (top left panel) and 42°C (top right panel); and protection of MDH at 37°C (bottom left panel) and shrimp alkaline phosphatase (SAP) at 50°C (bottom right panel) by pure hexadecamers. Negative control (no chaperonin added), i.e., MDH, or SAP in bottom right panel, alone: blue diamond. The results shown are mean values (±SD) of triplicate experiments. The loss of enzymatic activity caused by heat was impeded or considerably attenuated by the wild type and Pf-H molecules. In contrast, Pf-R did not have this protective effect, except when purified hexadecamers were used. Source: Min et al., 2014.

**Figure 8:**
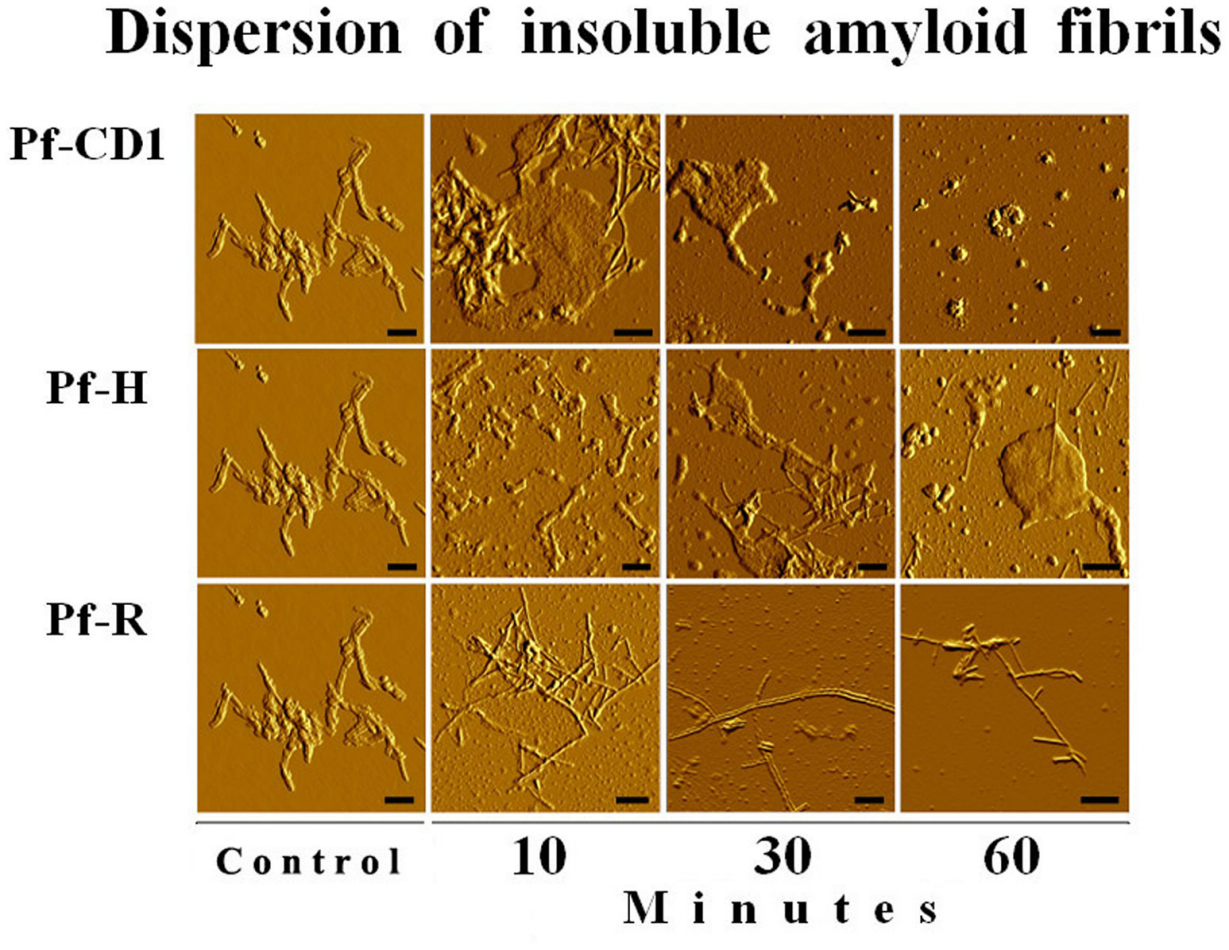
**The pathogenic mutant fails to disperse amyloid fibrils**. Dispersion of amyloid fibrils by archaeal Pf-CD1 (top row of panels), partial dispersion by Pf-H (middle row of panels), and no dispersion by Pf-R (bottom row of panels). Atomic Force Microscopy (AFM) of bovine insulin amyloid fibrils treated with Cpn and Mg++, and ATP. Control panels, no added chaperonin. Scale bar: 250 nm. Source: Min et al., 2014.

We found that Pf-R had very low ATPase activity by comparison with Pf-CD1 and Pf-H (Figure 6). Likewise, mixed oligomers of Pf-R were deficient in protecting test enzymes from heat denaturation (Figure 7), and were incapable of dispersing amyloid fibrils in contrast to Pf-CD1 and Pf-H (Figure 8). Remarkably, purified hexadecamers from the three molecules had similar enzyme protection capability; namely, hexadecamers formed by Pf-R had a chaperoning ability comparable to that of Pf-CD1 and Pf-H (Figure 7). The summary of results obtained with assays aimed at measuring the chaperoning capacity of the mutant Pf-R are displayed in Table 4. In conclusion, the Arg mutation affects the formation and stability of functional hexadecamers. Indeed, in native PAGE, Pf-R appears predominantly as oligomers smaller than hexadecamers and as monomers in contrast to Pf-CD1 and Pf-H (Min et al., 2014).

**Table 4:** **Summary of results showing the functional performance of the pathogenic mutant Pf-R in comparison with non-pathogenic counterparts^**a**^**.

**Gene**	***E. coli* exp**.	**Mixed oligomers**				**Hexadecamers**			
		**Heat res**.	**ATPase**	**Disperse fibrils**	**Form hexa-decamers**	**Protection**			
						**MDH**		**MDH**	**SAP**
						**37°C**	**42°C**	**37°C**	**50°C**
Pf-CD1	100	100	100	100	100	100	100	100	100
Pf-H	100	100	100 or less	100 or less	100 or less	100	100	100	100
Pf-R	100	100	Low	Low	Low	Low	Low	100	100

a *Source: Min et al., 2014*.

*exp, expression; res, resistance; 100, optimal; MDH, malate dehydrogenase; SAP, shrimp alkaline phosphatase*.

It is possible that some part of the effects observed using Pf-R by comparison with PF-CD1 were magnified somewhat because in the archaeal mutant the wild type amino acid, Ile, is hydrophobic and the mutant amino acid, Arg, is charged, whereas in the human situation the two amino acids, wild type and mutant, are charged, i.e., they are similar in this regard. For this reason, we generated and characterized the Pf-H mutant as control for any change that this charged residue might bring to the stability of the complex, which turned out to be minimal (Min et al., 2014).

We could not yet test isolated monomers because they tend to oligomerize rapidly in solution. However, *in silico* analysis by molecular dynamics simulations revealed that Pf-R monomers are rigid at the physiological temperature whereas those of Pf-CD1 are flexible; namely, the Ile138Arg mutation causes a loss of flexibility in the chaperonin molecule, which is also the result we obtained for the human mutant CCT5 (Min et al., 2014).

Experimentally, by applying quantitative calorimetry, current experiments are showing that the Pf-R hexadecamers are less resistant to heat and dissociate faster and at lower temperatures as compared with Pf-CD1.

In conclusion, the use of the archaeal chaperonin provided information that reflects the anomalies of the human mutant CCT5, which has been studied *in vitro* (Sergeeva et al., 2014). In a pioneering study, the human CCT4, and CCT5 subunits have been shown to form homohexadecamer complexes after expression of the subunits in *E. coli* (Sergeeva et al., 2013). The chaperoning ability of wild type CCT4 and CCT5 was tested in comparison with CCT4 C450Y and CCT5 H147R mutants. The functional tests included the suppression of aggregation of γD-crystallin and mutant huntingtin, and the refolding of beta-actin. In all tests the mutant subunits formed complexes with slightly deficient folding activities compare to the wild-type counterpart, although these effects were minimal in the case of CCT5 H147R. Both of the mutants produced were able to form complexes, although CCT4 C450Y showed limited stability and formed fewer megadalton-size complexes than CCT5 H147R. This study reinforces our conclusion that the biochemical effects of human mutations affecting the CCT5 complex, yet permitting viability, must of necessity be slight, attesting to the central role that we believe this complex plays in human protein homeostasis and physiology.

## Perspectives for the future

In the particular case discussed above, future research ought to be directed to the development of agents, e.g., chemical compounds, with the ability to interact with the mutant chaperonin molecule and restore its proper, functional configuration and thus, boost its capacity to form stable hexadecamers. The screening of compounds and preclinical testing could utilize the same *P. furiosus* experimental model used to reveal the abnormal features of the mutant CCT5 molecule. The same approach would apply to chaperonopathies involving other mutations in CCT5 that might be discovered, or mutations in any other subunit. Likewise, the *P. furiosus* model, as well as any other based on the archaeal and bacterial organisms discussed here, could be standardized and applied to elucidate the abnormalities of pathogenic chaperone molecules due not only to mutations but also to post-translational modifications. For example, Group III chaperonins could be used for modeling defects in human chaperonins involving the nucleotide sensing domain which, as mentioned earlier, is divergent in the canonical and non-canonical orthologs.

## Author contributions

ECdeM conceived the idea and outlined the project, participated in literature and databases searches, gathering and critical analysis of data, preparation of materials, and writing the manuscript. FTR participated in gathering of data and preparation of materials and writing the portions pertinent to chaperonins of Group III. AJLM participated in the outlining of the project and in literature and databases searches, gathering and critical analysis of data, preparation of materials, and writing the manuscript.

### Conflict of interest statement

The authors declare that the research was conducted in the absence of any commercial or financial relationships that could be construed as a potential conflict of interest. The reviewer LB and handling Editor declared their shared affiliation, and the handling Editor states that the process nevertheless met the standards of a fair and objective review. The reviewer MMM and handling Editor declared their shared affiliation, and the handling Editor states that the process nevertheless met the standards of a fair and objective review.
